# A Genome-Scale Metabolic Reconstruction of *Phytophthora infestans* With the Integration of Transcriptional Data Reveals the Key Metabolic Patterns Involved in the Interaction of Its Host

**DOI:** 10.3389/fgene.2018.00244

**Published:** 2018-07-10

**Authors:** David Botero, Iván Valdés, María-Juliana Rodríguez, Diana Henao, Giovanna Danies, Andrés F. González, Silvia Restrepo

**Affiliations:** ^1^Laboratory of Mycology and Phytopathology (LAMFU), Department of Biological Sciences, Universidad de los Andes, Bogotá, Colombia; ^2^Department of Design, Universidad de los Andes, Bogotá, Colombia; ^3^Group of Product and Process Design, Department of Chemical Engineering, Universidad de Los Andes, Bogotá, Colombia

**Keywords:** context-specific models, flux balance analysis, plant-pathogen interaction, hemibiotrophy, metabolic reconstruction, *Phytophthora infestans*

## Abstract

*Phytophthora infestans*, the causal agent of late blight disease, affects potatoes and tomatoes worldwide. This plant pathogen has a hemibiotrophic lifestyle, having an initial biotrophic infection phase during which the pathogen spreads within the host tissue, followed by a necrotrophic phase in which host cell death is induced. Although increasing information is available on the molecular mechanisms, underlying the distinct phases of the hemibiotrophic lifestyle, studies that consider the entire metabolic processes in the pathogen while undergoing the biotrophic, transition to necrotrophic, and necrotrophic phases have not been conducted. In this study, the genome-scale metabolic reconstruction of *P. infestans* was achieved. Subsequently, transcriptional data (microarrays, RNA-seq) was integrated into the metabolic reconstruction to obtain context-specific (metabolic) models (CSMs) of the infection process, using constraint-based reconstruction and analysis. The goal was to identify specific metabolic markers for distinct stages of the pathogen's life cycle. Results indicate that the overall metabolism show significant changes during infection. The most significant changes in metabolism were observed at the latest time points of infection. Metabolic activity associated with purine, pyrimidine, fatty acid, fructose and mannose, arginine, glycine, serine, and threonine amino acids appeared to be the most important metabolisms of the pathogen during the course of the infection, showing high number of reactions associated with them and expression switches at important stages of the life cycle. This study provides a framework for future throughput studies of the metabolic changes during the hemibiotrophic life cycle of this important plant pathogen.

## Introduction

Late blight has been a major threat to global food security ever since the Irish potato famine of the mid-nineteenth century (Fry, 2008). Globally, late blight costs billions of dollars annually. Management mostly involves the use of fungicides, resistant host genotypes, and cultural procedures designed to reduce the introduction, survival, or infection rate of the causal organism, *Phytophthora infestans* (Fry, 2008). The pathogen's genome plasticity, with the presence of transposable elements and repeat-rich regions, fosters the emergence of a high number of mutations which increases the probability of overcoming major resistance genes and evolving to counteract other control methods, such as fungicide applications (McDonald and Linde, 2002; Fry, 2008).

*Phytophthora infestans* is a hemibiotrophic pathogen having an initial biotrophic phase, followed by a necrotrophic phase during which host cell death is induced (Fry, 2008). The first stages of infection are asymptomatic because of effective suppression of the plant's immune response caused by the secretion of proteins known as effectors that can change the host's physiology and facilitate colonization. The “shift” between the biotrophic and necrotrophic phases is poorly understood and is becoming an important field of study. Lee and Rose (2010) proposed a hypothetical model of transition between biotrophy and necrotrophy where effectors play an important role; for example, SNE1 acts by suppressing programmed cell death in the host and halting the effects of necrosis inducing proteins (NIPs) (Kelley et al., 2010) until the necrotrophic phase is initiated. This “simultaneous accelerator and brake model” (Lee and Rose, 2010) strategy of infection could explain the onset of necrosis and provides clues for the regulation of pathogenesis as well as the organism's lifestyle.

Previous studies have used metabolic networks as tools to elucidate the lifestyle and life cycle of particular plant pathogens (Duan et al., 2013). In a broad sense, a metabolic model is a holistic view of a system, and it represents the connectivity between metabolites and reaction-catalyzing enzymes (Pitkänen et al., 2010). A metabolic network could be reconstructed in a top-down manner taking advantage of the organism's genome annotation to extract critical information on the protein elements involved in metabolism. These models are often called genome-scale reconstructions and can gather and integrate all the available information for complex systems, such as host-pathogen interactions. With increased amounts of information on the physiology, biochemistry, and genetics of the target organism, the predictive capacity of the model is greatly improved (Pitkänen et al., 2010).

There are many types of interaction networks, including protein–protein, metabolic, signaling, and transcription-regulatory networks. None of these networks are independent (Barabási and Oltvai, 2004), and together, they contribute to the understanding of a system. In 2013, Seidl et al. (2013) constructed a gene network of *P. infestans* and determined functional models related to its life stages using predictions of protein-protein interactions. Rodenburg et al. (2017) then reconstructed the first genome-scale metabolic model for *P. infestans*, based on reactions found in the Kyoto Encyclopedia of Genes and Genomes (KEGG) (Rodenburg et al., 2017). They also used RNA sequencing data to quantify gene expression in four *in vitro* life stages of *P. infestans*, i.e., mycelium, sporangia, zoospores, and germinating cysts (Rodenburg et al., 2017).

However, until now, the interaction of the pathogen and its host has not been studied *in silico*. The novelty of our study was the use of a genome-scale metabolic network of *P. infestans* and the derived context-specific models (CSMs) of the infection process to identify important metabolic markers in the pathogenic phases of this oomycete. Furthermore, we tested the hypothesis of a significant change during infection at the level of metabolism. The omics data selected for this study responded to the criteria of quality and comprehensiveness. The genomics data of the T30-4 *P. infestans* strain were chosen for the analysis because it represents the best annotated genome for the species (Haas et al., 2009). The transcriptomic data of the strain 1,306 were selected for the present study because it is the most comprehensive study of the metabolic changes of the pathogen during its development (Ah-Fong et al., 2017). For the initial genome-scale metabolic network, we used our own reconstruction, different from the one proposed by Rodenburg et al. (2017) because when they published their reconstruction, ours was in an advanced stage and we decided to continue using it. A thorough comparison of both reconstructions is made available in this study to the scientific community.

## Materials and methods

### Metabolic reconstruction

A draft metabolic reconstruction of *P. infestans* was generated based on the *P. infestans* proteome available on UniProt Knowledgebase (UniProtKB:UP000006643) (Haas et al., 2009; The UniProt Consortium, 2015). This proteome was annotated using the Automatic Annotation Server (KAAS) (Moriya et al., 2007), in order to obtain the enzyme codes (EC) associated with each protein. Using these ECs, the reaction list for *P. infestans* was retrieved from the KEGG (Ogata et al., 1999). The list of families of transport proteins reported for *P. infestans* were retrieved from TransportDB (Ren et al., 2007) and were manually curated. Finally, the directionality of each reaction was assigned using Gibbs free energy values provided by the database of MetaCyc (Karp et al., 2002).

Once a draft of the metabolic reconstruction was obtained, 250 dead-end metabolites were identified using GapFind algorithm (Satish Kumar et al., 2007). Then GapFill and multiple databases were used to solve problematic metabolites: KEGG database, transport reactions of *BiGG* database (Schellenberger et al., 2010), and transport reactions of BioModels (BioModels, 2012). Finally, the pathways related with every set of reactions were retrieved from KEGG. The model was not compartmentalized because this new level of complexity produced a non-functional model that cannot grow in minimal media when it was tested.

The metabolic reconstruction was modeled using flux balance analysis (FBA) and flux variability analysis (FVA) (Feist and Palsson, 2010; Orth et al., 2010; Lakshmanan et al., 2014). The reconstructions and FBA were conducted using COBRA Toolbox 2.0 (Schellenberger et al., 2011) following the procedures suggested by Thiele and Palsson (2010) and Schellenberger et al. (2011). The simulations for the full model were performed in Henninger minimal media (Henniger, 1963). Biomass was used as objective function for simulations. The biomass reaction was defined using as a reference the objective function from the metabolic model of *Toxoplasma gondii* (Song et al., 2013). Stoichiometric coefficients were taken from the *T. gondii* model but only metabolites present in the *P. infestans* model were included (Table S1).

### Construction of context-specific models and metabolic simulations

Context-specific models (CSMs) of the infection process for *P. infestans* 06_3928A and T30-4 strains were generated using microarray data available in the Gene Expression Omnibus (GEO) database as the primary source of information (Table 1).

**Table 1:** Data used for the formulation of Context Specific Models (CSMs).

**Phase /treatment**	**Graphical representation**	**GEO Accession number (*Phytophthora infestans* isolate used)**	**References**
Mycelia on rye sucrose agar	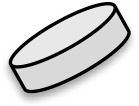	GSE33240 (Average expression of isolate 06_3928A)	Cooke et al., 2012
2 days post inoculation on potato leaflets	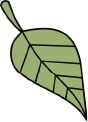		
3 days post inoculation on potato leaflets	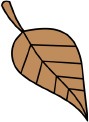		
4 days post inoculation on potato leaflets	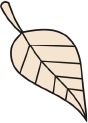		
Mycelia on rye sucrose agar	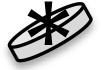	GSE14480 (Isolate T30-4)	Haas et al., 2009
2 days post inoculation on potato leaflets	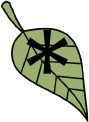		
3 days post inoculation on potato leaflets	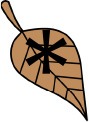		
4 days post inoculation on potato leaflets	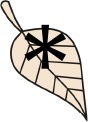		
5 days post inoculation on potato leaflets	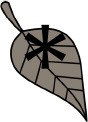		

*GEO Accession numbers are provided as well. A graphical representation of each stage is provided for reference*.

The TIGER toolbox (Jensen et al., 2011) was used to integrate transcriptional data (microarrays, RNA-seq) into metabolic reconstructions. This was done by mapping expressed genes to enzymes that catalyze each metabolic reaction (known as gene-protein reactions) and by generating a series of mathematical inequalities. The Limma R package (Smyth, 2005) was used for the microarray analysis, and the metabolic adjustment by differential expression (MADE) algorithm was used to integrate the transcriptomic data and metabolic reconstructions (Jensen and Papin, 2011; Jensen et al., 2011). The MADE algorithm requires two or more sets of microarray data to create a sequence of binary expression states to reconstruct the reactions, hence no thresholding is needed. Then, the TIGER toolbox (Jensen et al., 2011) sets constraints for the optimization problem, yielding one individual metabolic model for each condition or context-specific models (CSMs). For the FBA, the MADE algorithm only considers the reactions that were tagged as ON in the previous step. A gene/reaction pair that is “ON” in the ON-OFF network does not necessarily show a flux reaction in the FBA network. With these two matrices, networks (CSMs) were built and analyzed.

### Comparisons of FBA for all CSMs and comparison of *P. infestans* strains 06 _3928A and T30-4 CSMs

In order to compare the different strains at different days post inoculation, FBA for every CSM was calculated. For every FBA of each CSM the active reactions (flux different from zero, in mmol/gDW/h) were established. Then based on those active reactions, calculations of pan, core, accessory, and unique reactomes were performed. The definitions for each one is as follows: (i) Pan reactome: reactions (and pathways for the reactions) present in all CSMs compared; (ii) Core reactome: reactions shared among the CSMs compared; (iii) Accessory reactome: reactions present in more than one CSM but not in all; and (iv) Unique reactome: reactions present in only one CSM. From now on, when unique, core and accessory are referred in the context of metabolic pathways, it does not imply that a whole pathway is unique, accessory or core, but it means that the reactions found in each set belonging to these pathways could be unique, accessory or core. It is a way to contextualize the reactions at the level of metabolic pathways.

**Figure 1:**
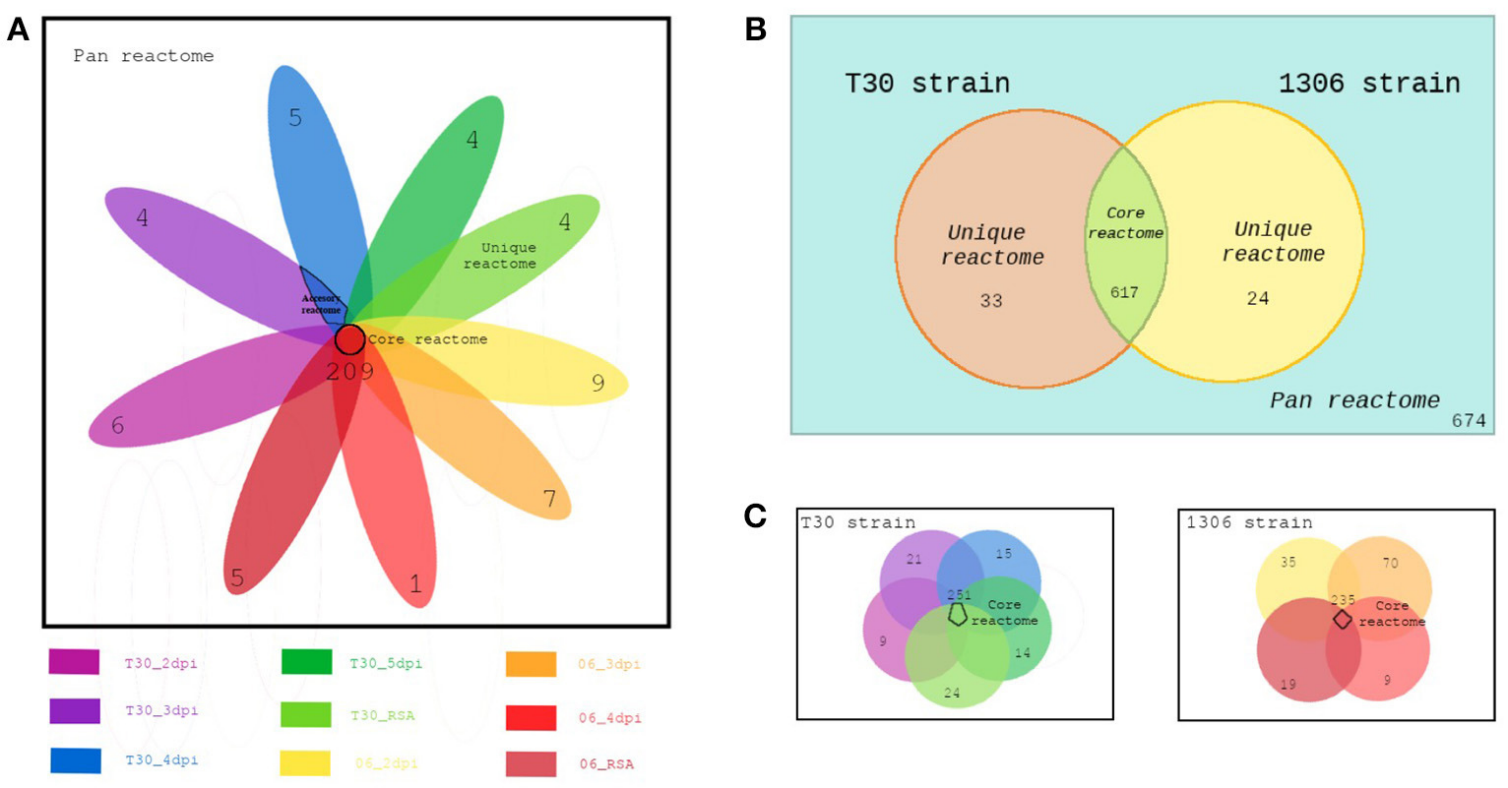
Comparisons of CSMs at different levels **(A)** 9 CSMs **(B)** between strains **(C)** within the strains.

These calculations were performed at three levels. The first level, evaluates the whole model based on the reactions that are active in each strain using Venny 2.1.0 (Oliveros, 2016) to produce the values of a Venn diagram with the shared and unique reactions between the two models (two strains). The second level evaluated the general behavior at the metabolic level of the nine CSMs. The third level assesses the metabolic activity at the strain level at different time points. In this comparison, all the CSMs were grouped into two sets for each strain. For example, the pan reactome of every strain consists of the reactions that were active in any stage of development (dpi) for a particular strain. The core reactome of the strains is the reactions shared by the CSMs of each strain and the core of cores. The reactions shared by the two core reactomes, was also calculated. Finally, the accessory and unique reactomes for each strain were obtained. Figure 1 illustrates the concepts of the comparisons.

### Hierarchical clustering heatmap

In order to uncover the relationships among the different CSMs we performed a hierarchical clustering of CSMs separated by strain. Z-scores to normalize flux values (mmol/gDW/h) were computed. The linkage matrix with Euclidean distance based on Ward's method was calculated (Ward, 1963). A dendrogram was done for coloring purposes using a color threshold of 0.25. Finally, cluster maps of the two strains were calculated. All procedures were performed in Python using SciPy package.

## Results

### Metabolic reconstruction

**Figure 2:**
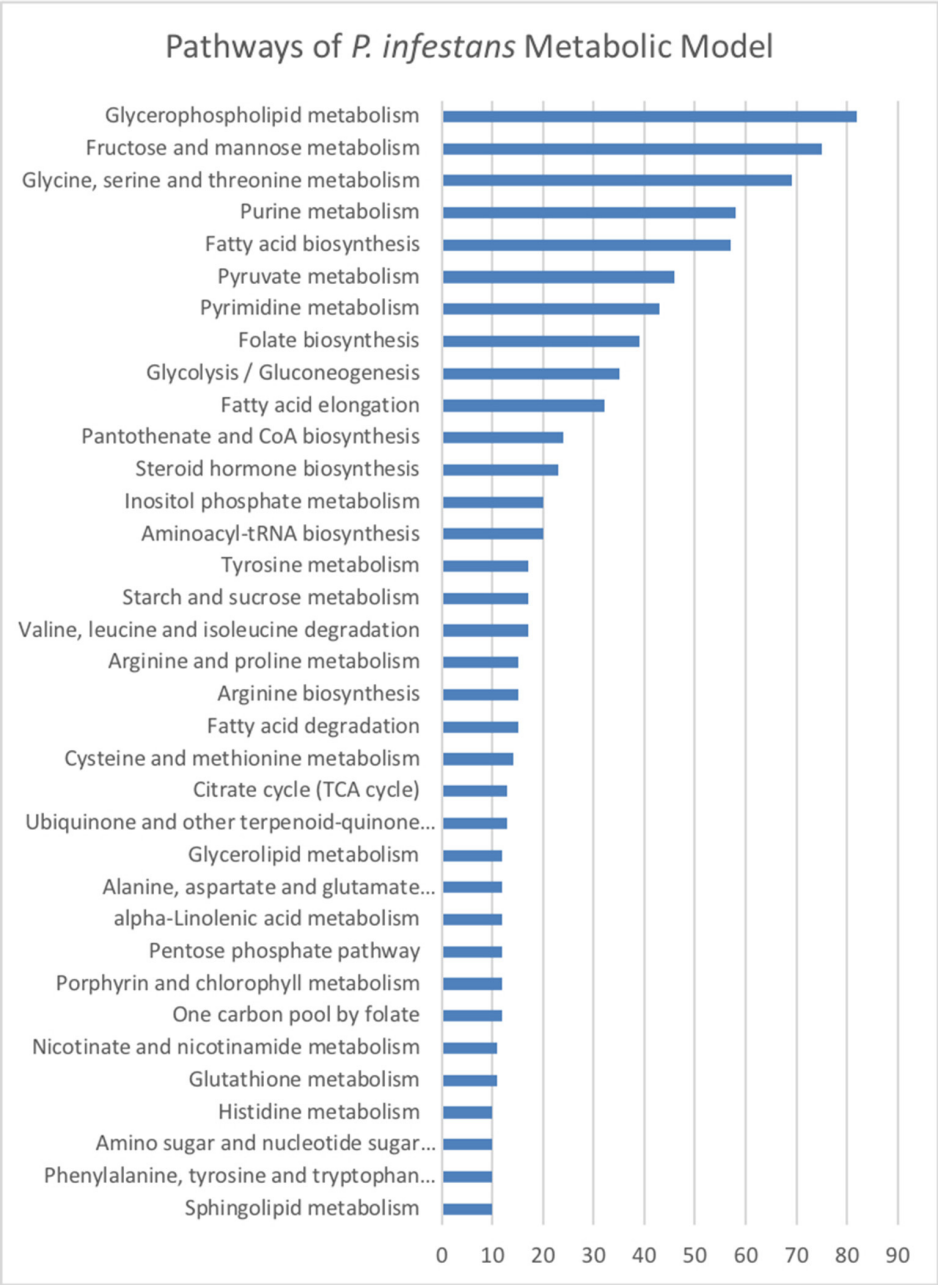
Reactions of the metabolic reconstruction of *Phytophthora infestans* grouped by metabolic pathways according to the Kyoto Encyclopedia of Genes and Genomes (KEGG). Number of reactions by category are listed.

The final reconstruction of *P. infestans* accounted for 1,530 metabolites participating in 1,571 reactions (Table 2) belonging to 1,094 pathways (Table S2) and associated to 1,375 genes; 194 reactions are orphan (without an associated gen). The 35 exchange reactions represent the metabolites of the Henninger minimal media for simulation of the complete model. Furthermore, 249 reactions participating in transport were included in contextual models. Transport reactions were selectively chosen by MADE based on transcriptomic data in order to simulate different metabolite exchanges in every stage. The genome of *P. infestans* T30-4 strain has 19,934 genes, so, the metabolic genes (1,375) cover 7% of the coding gene space. The pathways with more reactions in the reconstruction of *P. infestans* were associated with glycerophospholipid metabolism, then fructose and mannose metabolism, and glycine, serine, and threonine metabolism (Figure 2, Table S2). The list of top 18 pathways and the number of reactions by category is shown in Figure 2. The whole list is shown in Table S2.

**Table 2:** Metabolic reconstruction of *Phytophthora infestans*.

***Phytophthora infestans*** **metabolic reconstruction properties**	
GPR	1375
Reactions	1571
Cellular	1287
Transport	249
Exchange reactions	35
Metabolites	1530

*GPR, Gene protein reaction associations*.

### Flux balance analysis of nine different context-specific models (CSMs)

Using the MADE algorithm, nine CSMs were obtained and analyzed under the objective function biomass production (Tables S8–S10). The nine CSMs correspond to the two strains growing on media and at different time points during the infection: 06_3928A (mycelia on rye sucrose agar (RSA), 2, 3, and 4 days post-inoculation [dpi]) and T30-4 (mycelia on RSA and 2, 3, 4, and 5 dpi; Table 1).

**Figure 3:**
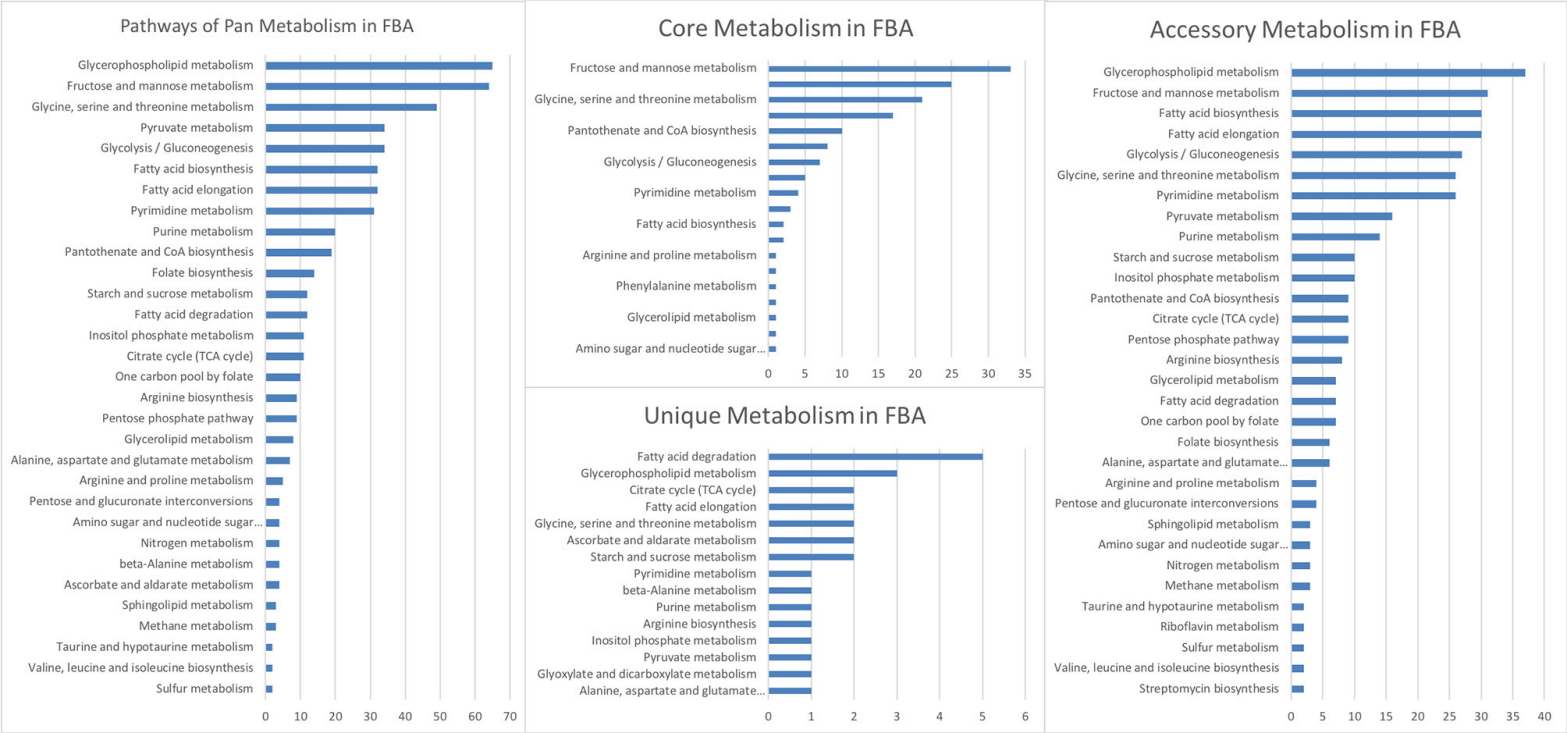
Reactions of the metabolic reconstruction of *Phytophthora infestans* grouped by metabolic pathways according to the Kyoto Encyclopedia of Genes and Genomes (KEGG) for pan, core, accessory, and unique reactomes calculated over the nine context-specific (metabolic) models (CSMs).

After the analysis of the model, a second level of analysis was done to compare all CSMs, by pooling the data for the two strains and the treatments. A total of 596 (86.1%) reactions were obtained for the two strains (core reactions) while the number of pathways in the core model was 144 (Table S2). The pathways with more reactions assigned in the core corresponded to the fructose and mannose metabolism, suggesting that this metabolism is very important for the pathogen to survive in its host. The analysis showed 26 unique (Table S2) and 366 accessory pathways for both strains and treatments combined. Accessory pathways are the ones present in more than one CSM (growth medium or during infection) but not in all. In other words, the accessory pathways represent the metabolic plasticity of the strains, rapidly adapting to a changing environment. The pathways with more reactions in the accessory group corresponded to glycerophospholipid metabolism, fructose and mannose metabolism, fatty acid biosynthesis, glycolysis—gluconeogenesis, amino acid and purine—pyrimidine metabolisms (Table S2). A total of 26 pathways were unique to one CSM (Table S2). The pathways with more reactions in the unique category were the ones involved in fatty acid degradation (Figure 3, Table S2). They were unique in the CSMs at early time points during plant infection, in both strains (Table S2). The specific reactions involved in the fatty acid degradation were the KEGG reactions: R00631, R03857, R03990, R04751, and R04754. In the CSMs corresponding to the growth in RSA medium, the unique pathways corresponded to the purine and pyrimidine metabolism, as well as amino acids and starch/sucrose metabolism (Table S2).

### Flux balance analysis of two *Phytophthora infestans* strains growing in medium and in the host

The two *P. infestans* strains, 06_3928A and T30-4, were compared against each other. Table 3 shows the active fluxes (mmol/gDW/h) for each strain and each time point, as well as the core for each strain and for both strains. For all the treatments pooled, a total of 617 (91.54%) core reactions, 24 (3.56%) unique reactions for strain 06_3928A, and 33 (4.90%) unique reactions for strain T30-4, respectively, were identified (Table S3). The core pathways correspond to the pathways in common for the two strains in all the treatments together.

**Table 3:** Comparisons of active fluxes (mmol/gDW/h) in *Phytophthora infestans* strains T30-4 and 06_3928A when growing on rye sucrose agar (RSA) medium and during the time course of infection.

**Strain**	**RSA**	**2 dpi**	**3 dpi**	**4 dpi**	**5 dpi**	**Pan**	**Core of each strain**	**Core**
*P. infestans* 06_3928A	389	503	513	330	NA^*^	641	235	212
*P. infestans* T30-4	489	495	503	501	337	650	251	

* *This time point was not studied for the P. infestans 06_3928A strain and therefore the data are not available in the GEO database. dpi, days post inoculation*.

In order to characterize each stage of development for each strain, the pan and core reactomes as well as the accessory and unique pathways were retrieved for each strain from the different treatments, for the nine CSMs (Table S4). In this case, data were analyzed within each strain and not between them, for example, the core reactions for the T30-4 strain are the ones present in all the CSMs (*n* = 5: five treatments: RSA and four time points) for that strain.

The pathways with more reactions active in FBA for each strain are depicted in Figure S1 (pan and core reactomes) and Figure S2 (accessory and unique reactomes). In the case of the pan and core reactomes, the list of pathways showed the similarity between the two strains, reflecting a common metabolism. For both the core and the pan, the three pathways with more reactions corresponded to fructose and mannose, glycerophospholipid, and glycine—threonine metabolisms (Figure S1, Table S4). In the case of the accessory and unique reactomes, the two strains also showed a common set of metabolisms. The metabolic category with more reactions for the accessory pathways was the fatty acid biosynthesis and for the unique, the fatty-acid elongation pathways (Figure S2, Table S4).

The unique pathways and reactions of each strain reflect the metabolic versatility of each strain. In this analysis, the unique pathways correspond to pathways that are only active in one treatment. Also, unique can be additional reactions that the pathogen is manipulating (with an active flux) to thrive in the treatment under study. Table S4 shows the list of unique reactions for the models of the two strains, the treatment in which they were unique, as well as the flux in each of the treatments. Some of the most interesting pathways related with the unique reactions were some involved in the nitrogen and the ß-alanine metabolisms only present in the RSA medium and not *in planta* (Table S4). Furthermore, some reactions of the fructose and mannose metabolism that are highly represented *in planta* condition, are active when *P. infestans* strain T30-4 is growing on medium and at the latest time point of infection (5 dpi). Some of the reactions had positive while others had negative fluxes (mmol/gDW/h) implying a different directionality of the reactions (Table S4).

### Markers of life stages in the host

**Figure 4:**
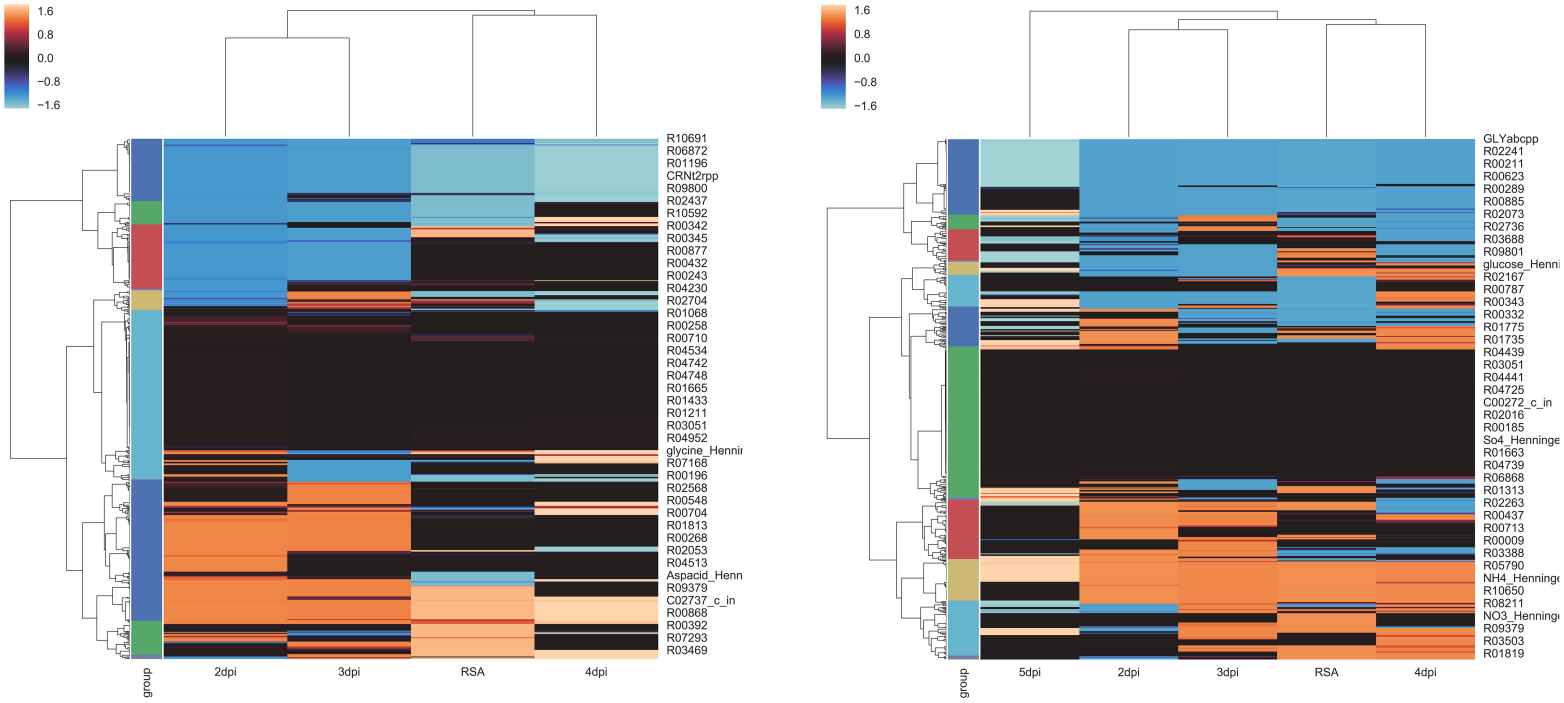
Hierarchical Clustering Heatmaps of *Phytophthora infestans* strains: **(A)** strain 06_3928A and **(B)** strain T30-4. Z-scores to normalize flux values were computed (mmol/gDW/h). Linkage matrix with Euclidean distance based on Ward's method was calculated. A dendrogram was calculated for coloring purposes using a color threshold of 0.25. Finally, cluster maps of the two strains were calculated.

Hierarchical clustering heatmaps for *P. infestans* strains are shown in Figure 4. Clustering of the time points showed the same results for both strains. Earlier time points (2 and 3 dpi) grouped together and apart from later time points (4 and 5 dpi). In both strains, the metabolism 4 dpi was the most similar to the metabolism of the strains growing in the RSA medium. From Figure 4 it is also evident that the metabolism did not dramatically change over the course of infection. The later time points, 5 dpi for strain T30-4 and 4 dpi for strain 06 _3928A, were the most different.

Four groups of reactions were identified. The host group corresponded to those reactions that are not active in RSA medium and become active at early time points after inoculation (Table S5). The biotrophy group are the reactions that are active in the first stages of infection (2–3 dpi) and decrease at the later stages of infection (4 dpi). The necrotrophy group are the reactions that are inactive during the first stages of infection (2–3 dpi) and increase at the later stages of infection (4–5 dpi). Finally, the transition group are the reactions with changes between 2 and 3 dpi, and 3 and 4 dpi. The changes are defined as fluxes that change from absolute values smaller than 100 to >500 mmol/gDW/h; in several cases the reactions were abruptly switched on (absolute flux value of 1,000 mmol/gDW/h) or off (flux value of 0) in this group. Table S5 summarizes the reactions in the four groups.

When the pathogen is inoculated, as expected, several reactions of the metabolism are altered (their metabolic fluxes changed). The pathways with more reactions associated belonged to the purine and pyrimidine metabolism, the glycerophospholipid metabolism, and some reactions involving isomerase enzymes of the glycolysis/gluconeogenesis metabolism (Table S5). In the case of the pyrimidine metabolism, three transferases showed active fluxes at early time points in the two strains: phosphate adenylyltransferase, phosphate uridylyltransferase, and phosphate cytidylyltransferase.

The biotrophy phase of infection (2–3 dpi) did show some interesting metabolic markers. First, there was a change in the biosynthesis of some amino acids and the synthesis of 1,3-beta-glucan, the main component of the *Phytophthora* genus' cell walls (Bartnicki-Garcia and Wang, 1983; Table S5).

In the necrotrophy phase of infection, the glycerophospholipid metabolism was altered, their metabolic fluxes changed, but this time with more active fluxes than in the host category (Table S5). For the transition between biotrophy and necrotrophy, the metabolic markers of this phase corresponded to folate biosynthesis, the use of sugars, in particular fructose and mannose, and also nitrogen metabolism specifically, the metabolism (biosynthesis) of the amino acids, arginine, serine, glycine, and threonine (Table S5). The fatty acid-elongation pathways also showed active fluxes at early time points (biotrophy) and changed to flux 0 or the directionality of the reaction at later time points.

### Comparison of the metabolic reconstruction with a previous metabolic model of *P. infestans*

A comparison was made between the model of Rodenburg et al. (2017) (iSR1301) and the one produced in the present study to determine the similarities and differences between them (Table 4). For the full model reconstructions before CSMs reconstructions, 778 reactions within the cell were shared. Likewise, 794 unique reactions were identified for the model used in this study, and 708 for the model constructed by Rodenburg et al. (2017). Also, the pathways, in which the reactions shared between the two reconstructions are involved, were identified (Figure S3).

**Table 4:** Comparisons between the iSR1301 metabolic model of *P. infestans* (Rodenburg et al., 2017) and the one presented here.

**Feature**	**iSR1301**	**This study**
Reactions	2,394	1,571
Compounds	2,685	1,530
Genes	1,408	1,375
Compartments	Extracellular space, cytosol, mitochondria, endoplasmic reticule, Golgi, peroxisome and vacuole	Extracellular space and cytosol
Context-specific models	Mycelium, sporangium, zoospore and germination cyst	Comparison of T30-4 strain to 1306 strain at different times of infection: RSA, 2dpi,3dpi,4dpi, and 5 dpi

*RSA, Rye-sucrose Agar; dpi, days post inoculation*.

In the case of transport and exchange reactions, they were analyzed separately to determine the correspondence between them, because they did not have the same database nomenclature. Only a correspondence of 7 exchange and transport reactions between the two models was found. Additionally, when we analyzed in detail the reconstruction made by Rodenburg et al. (2017) we found that the extracellular compartment is disconnected from the rest of the reconstruction. In the category of transport reactions, we obtained 249 reactions between the extracellular and intracellular space, while in Rodenburg et al. (2017) only 17 reactions were obtained. A comparison of the number of shared and unique genes between the two models was also performed. Between the model constructed in this study and the model constructed by Rodenburg et al. (2017), a total of 781 genes were shared, and 564 and 520 unique genes were identified, respectively.

Finally, a comparison was made between the compounds that make up the objective function of both reconstructions. Tables S6, S7 include detailed information and a correspondence of the reactants and products of the objective functions of the two models. The model of Rodenburg et al. (2017) has an objective function composed of 31 metabolites and our objective function was composed of 39 metabolites. In total, the two models shared 25 compounds. Rodenburg et al. (2017) adopted stoichiometric coefficients of 1 for all the compounds of the objective function. As was mentioned before, in our reconstruction we used as base the coefficients of *T. gondii* (Song et al., 2013). The value of the objective function in the FBA for both models before the reconstruction of the CSMs was compared (22.41 and 4.99 h^−1^ for (Rodenburg et al., 2017) and our model, respectively). However, our model has bounded transport reactions while the Rodenburg et al. (2017) model has unbounded transport reactions. In order to made them comparable a calculation of growth rate using FBA was performed with unbounded transport reactions with a result value of 45.21.

The CSMs in the metabolic model published in 2017 included mycelia, sporangia, zoospores, and germinating cysts, which originally came from *in vitro* culture media. Our reconstructed CSMs did not differentiate among the pathogen's structures but analyzed different life stages of the pathogen *in planta*.

## Discussion

This is the first metabolic reconstruction of *P. infestans* that used transcriptional data to create models (context- specific models, CSMs) of several life stages of this oomycete inside the host. The CSMs represent the subset of reactions from the genome-scale metabolic model (GSM) that show fluxes that are active in a given context (Robaina Estévez and Nikoloski, 2015). In this study, the contexts were the rye-sucrose agar medium and the days after the inoculation on the host. Moreover, the models obtained here provide insights of the general metabolism of the oomycete during its growth, development, and pathogenesis. Therefore, this study contributes to the general knowledge of the causal agent of the late blight disease.

Tools (CSMs) for studying the pathogen are now available as well as metabolic markers in all the main life-cycle stages *in planta*. A previous study modeled a gene network of *P. infestans*, based on protein-protein data and identified gene models related to selected developmental stages (e.g., hyphae, sporangia, cleaving sporangia, zoospore, and cyst), but none of the stages of the infection process (infection on leaflets) were included (Seidl et al., 2013). The study of Rodenburg et al. (2017) proposed the first metabolic reconstruction of the pathogen and explored the metabolic response of this oomycete under several asexual life cycle stages. The network reconstruction in this study confirmed previous results and included the metabolic processes that occur during infection. Together these studies could lead to a complete picture of the pathogen's metabolism. For example, Rodenburg et al. (2017) showed the importance of fatty acids in *Phytophthora* zoospores. Fatty acid degradation (β-oxidation) is not active in the zoospore stage (Rodenburg et al., 2017). In this study, the importance of the fatty acids biosynthesis, elongation, and degradation pathways during the infection cycle was also shown. Therefore, extending the Rodenburg et al. (2017) study, the results of this study showed that shortly after the infection, some reactions related with the fatty acid elongation are activated.

Both reconstructions started with similar genomic data and used similar predictive software to obtain the list of reactions that constituted the whole reconstruction before the CSMs. Rodenburg et al. (2017) compartmentalized their reconstruction and then generated Context Specific Models (CSMs). However, they did not perform the Flux Balance Analysis (FBA) of the CSMs but compared them at the structural level. In contrast, we did not compartmentalize the metabolic reconstruction but conducted the FBA and the Flux Variability Analysis (FVA) of the CSMs. In Rodenburg et al. (2017) only asexual life stages of the pathogen were considered, while our analyses considered the plant infection up to 120 h allowing us to include the biotrophic and necrotrophic phase of the pathogen's life cycle. It is important to consider that the algorithm that we used to reconstruct the CSMs, MADE (Metabolic Adjustment by Differential Expression), is different to iMAT (Iterative Method with Adaptive Thresholding) used by Rodenburg et al. (2017). MADE relies on the dynamic changes in the level of expression of the genes associated to the reactions, thus, maintaining a coherence between all CSMs, whereas, iMAT (Interactive Method with Adaptive Thresholding) depends on a specific differential expression threshold within each biological stage analyzed.

The novelty of our work with regards to Rodenburg's from a biological point of view is the use of the modeling and analysis of the distribution of fluxes in our CSMs to understand the differences of the life stages during the plant's infection. Indeed, our CSMs cover the plant infection, the most important phase of a pathogen's life cycle, including the biotrophy necrotrophy life stage. Furthermore, we performed different comparisons between two strains and the life stages including the identification of pan, core, unique, and accessory reactomes through the modeling of the CSMs. These comparisons and modeling contributed to the understanding of the dynamics of pathogenicity under the brake-accelerator hypothesis (Lee and Rose, 2010). The putative biological markers of switching between biotrophy and necrotrophy can be used for the design of control strategies of the pathogen. Also, the understanding of that switch improves the scientific knowledge into the pathogenicity mechanisms.

This study presents a throughput tool for data mining the metabolism of the pathogen that is more appropriate than analyzing the genome. First, the publicly available genome (Haas et al., 2009) needs a re-annotation given that new studies show problems in some of its annotations. For example, Rodenburg et al. revealed one unannotated tyrosine decarboxylase gene and recommended to reassess the annotation of the genome (Rodenburg et al., 2017). Second, the construction of the CSMs allow for an understanding of the dynamics of the genome exploiting the data gathered in the databases and in most cases not thoroughly analyzed. Taking advantage of the CSMs, in this study strain-specific reactions or time point-specific reactions could be identified.

One of the hypotheses of this study, an overall metabolism change during the infection process, was confirmed since the overall pattern of the metabolic fluxes changed dramatically under the life stages analyzed. Furthermore, reactions involved in these changes were classified in different metabolic categories. This general result is in agreement with what was reported for the pathosystem *Septoria tritici—*wheat, where an RNA-seq analysis of the fungus showed that the most dominant set of transcripts were assigned to metabolic processes making up to 50% of the transcripts that clustered during the course of the infection (Yang et al., 2013). The most striking change in the metabolism is when the pathogen enters the necrotrophic phase.

The main objective of this study was to assess metabolic differences among growth conditions or life stages, to find unique representative metabolic markers that could be associated with important biological processes, such as living in the host, biotrophy, transition between biotrophy and necrotrophy, and necrotrophy. Despite the “uniformity” shown by the metabolism of *P. infestans* under different growth conditions, there were some unique reactions associated to each life stage model. These results support data from other researchers. For example, in another study, some amino acids were shown to be important in compatible interactions between potato and *P. infestans* (Grenville-Briggs et al., 2005). In the study reported here, the importance of the amino acid arginine for the biotrophic stage was demonstrated (active fluxes in the earlier time points). Arginine has been reported to be an important constituent of the pathogen effectors mainly expressed in biotrophy (Win et al., 2007).

This study revealed the importance of the fructose/mannose metabolism during infection of *Phytophthora* on its host. These metabolic pathways were the most or one of the most represented (number of reactions in these categories) in the core, pan, and accessory reactomes when the nine CSMs were pooled and/or when the strains were compared. Changes in the fructose concentrations in the host tissues have been previously shown (Judelson et al., 2009). Judelson et al. (2009) showed that fructose levels in the host increased three-fold and glucose doubled, during a 6-day infection period (Judelson et al., 2009). It is not known if the pathogen responds to these changes, but based on the results of this study, it can be suggested that fructose metabolism is also changing in the pathogen. The reaction catalyzed by the β-D-fructose 2,6-bisphosphate was active with a flux only *in planta*, when compared to growth on RSA medium in both strains (Table S5). This enzyme is a regulator with a dual function that allows it to control the rates of both glycolysis and gluconeogenesis (Buchanan et al., 2015).

Although most of the predicted fluxes by the models have been validated with experimental data, there are groups of fluxes showing inconsistencies between real and modeled data (Kjeldsen and Nielsen, 2008; Babaei et al., 2014). A main concern in FBA analyses is the choice of the objective function. Maximization of biomass is frequently used as the objective function; thus, the underlying hypothesis is that maximal growth rate is favored during evolution (Chen and Shachar-Hill, 2012). But efficiency does not always correlate with high growth rate (Schuster et al., 2008). Therefore, a variable objective function should be applied on each CSM reflecting biological evidence. For example, endogenous production of crucial, central metabolites should be enhanced during the biotrophic phase of hemibiotropic plant pathogens (Fernandez et al., 2013), while metabolities involved in nutrient scavenging should be enhanced during the necrotrophic phase. A good example of a metabolite that is changing during infection and could be used as an objective function is pyrimidine (García-Bayona et al., 2014). Given this context, metabolites that are used as precursors for the biosynthesis of amino acids and nucleotides, among others, should be the objective functions of the CSMs related to biotrophic phases, and metabolites corresponding to salvage pathways should be included in necrotrophic phases. This new approach could be the starting point for the generation of more accurate CSMs for *P. infestans*.

In conclusion, the metabolic reconstruction and the construction of CSMs provide important novel insights into the whole metabolism of *P. infestans* at different stages of development, growth, and pathogenesis of this plant pathogenic oomycete. For example, sugars like fructose, amino acids such as arginine, glycine, serine, threonine, and folate, among others, are metabolites that could influence successful host colonization. Network analyses allowed the development of a new tool to design new experiments to comprehend the complete metabolism of plant pathogens *in planta*. Despite the complexity of the metabolism of *P. infestans* at each life stage, a framework for future research that seeks to further understand the hemibiotrophic life cycle of *P. infestans* is now available. We are also aware that the use of two different strains can add some noise to the analyses but our approach at the pathway level permitted to assess some variation at the specific reactions used by each strain.

## Author contributions

SR, AG, IV, and DB designed the study. IV, DH, M-JR, and DB performed the analyses. All authors contributed to writing the manuscript and approved its final version.

### Conflict of interest statement

The authors declare that the research was conducted in the absence of any commercial or financial relationships that could be construed as a potential conflict of interest.

## Supplementary material

Figure S1 Reactions of the metabolic reconstruction of *Phytophthora infestans* grouped by metabolic pathways according to KEGG for pan and core reactomes calculated over each strain for every day post inoculation (dpi).

Figure S2 Reactions of the metabolic reconstruction of *Phytophthora infestans* grouped by metabolic pathways according to the Kyoto Encyclopedia of Genes and Genomes (KEGG) for accessory and unique reactomes calculated over each strain for every time point.

Figure S3 Comparisons between the iSR1301 metabolic model of *P. infestans* (Rodenburg et al., 2017) and the one presented here at the level of pathways of the reactions shared between the two models. Horizontal axis: number of reactions by pathway.

Table S1 Biomass composition for *P. infestans* objective function.

Table S2 List of pathways in the metabolic reconstruction. The pathways of core, pan, accessory and unique reactions for the two strains combined are also listed.

Table S3 List of core and unique reactions for the two models, strains 06_3928A and T30-4.

Table S4 Pan, core and accessory reactomes of each CSM.

Table S5 Specific reactions that showed a change in flux during the disease cycle (see text for more details on the selection).

Table S6 Comparison between the objective function of the model in this study and the published by Rodenburg et al. (2017), using the reactants of the objective function.

Table S7 Comparison between the objective function of the model in this study and the published by Rodenburg et al. (2017), using the products of the objective function.

Table S8 FBA for the 9 CSMS.

Table S9 Flux variability analysis of the 9 CSMs for the two strains; Flux values are expressed in mmol/gDW/h.

Table S10 Same as Table S9.

Supplementary Data *P. infestans*_M_model.xml. Metabolic model of *P. infestans* (sbml). Pinfestans_project.mat. Matlab project with full metabolic model, FBA, FVA, and context-specific models.
