# Supplementary figures and images for: A Genome-Scale Metabolic Reconstruction of Phytophthora infestans With the Integration of Transcriptional Data Reveals the Key Metabolic Patterns Involved in the Interaction of Its Host
Source: Front Genet. 2018 Jul 10;9:244. doi: 10.3389/fgene.2018.00244 (PMC6048221; doi:10.3389/fgene.2018.00244)

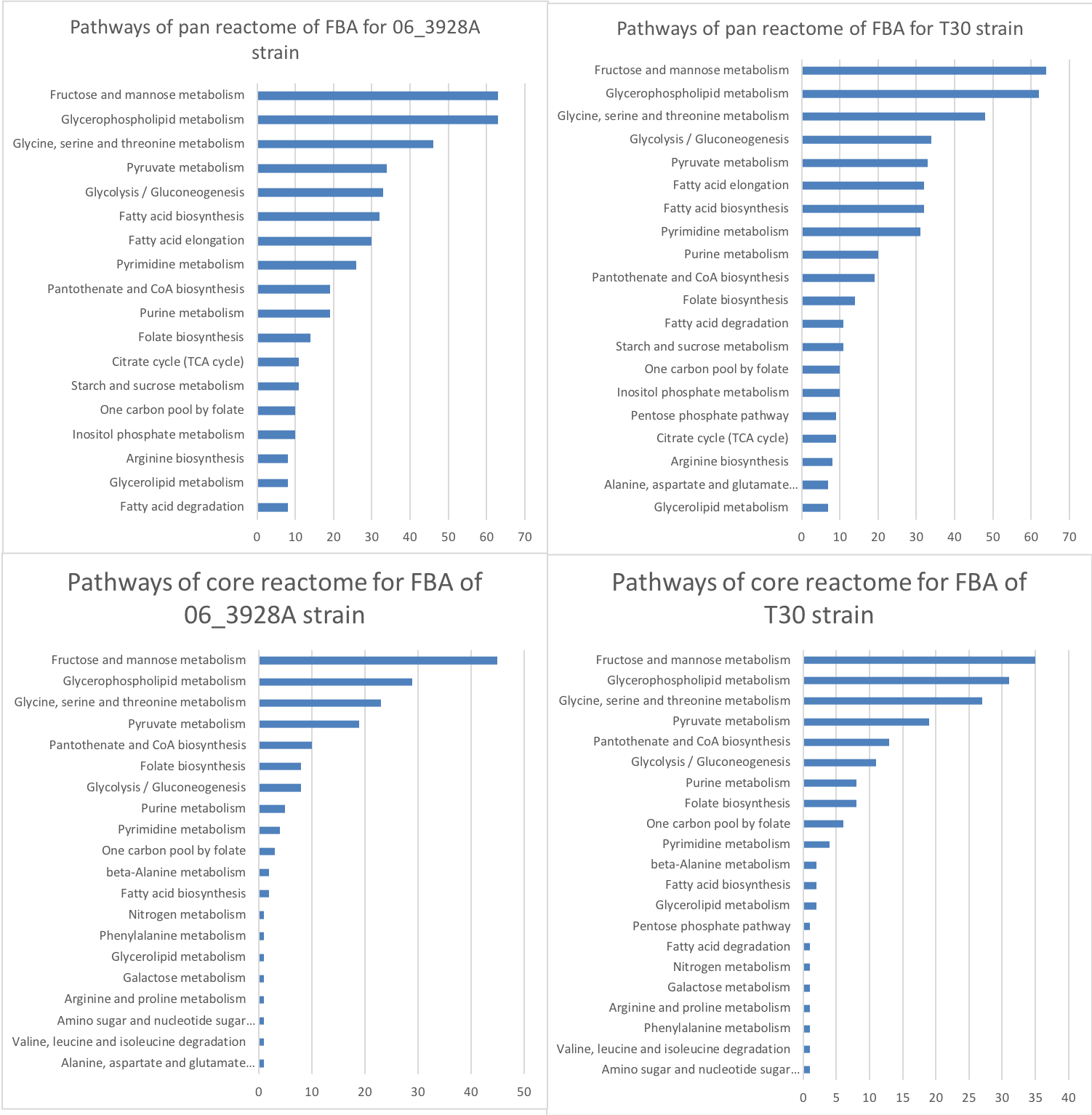

Supplement: Figure S1 — Reactions of the metabolic reconstruction of Phytophthora infestans grouped by metabolic pathways according to KEGG for pan and core reactomes calculated over each strain for every day post inoculation (dpi). [file Image_1.JPEG]

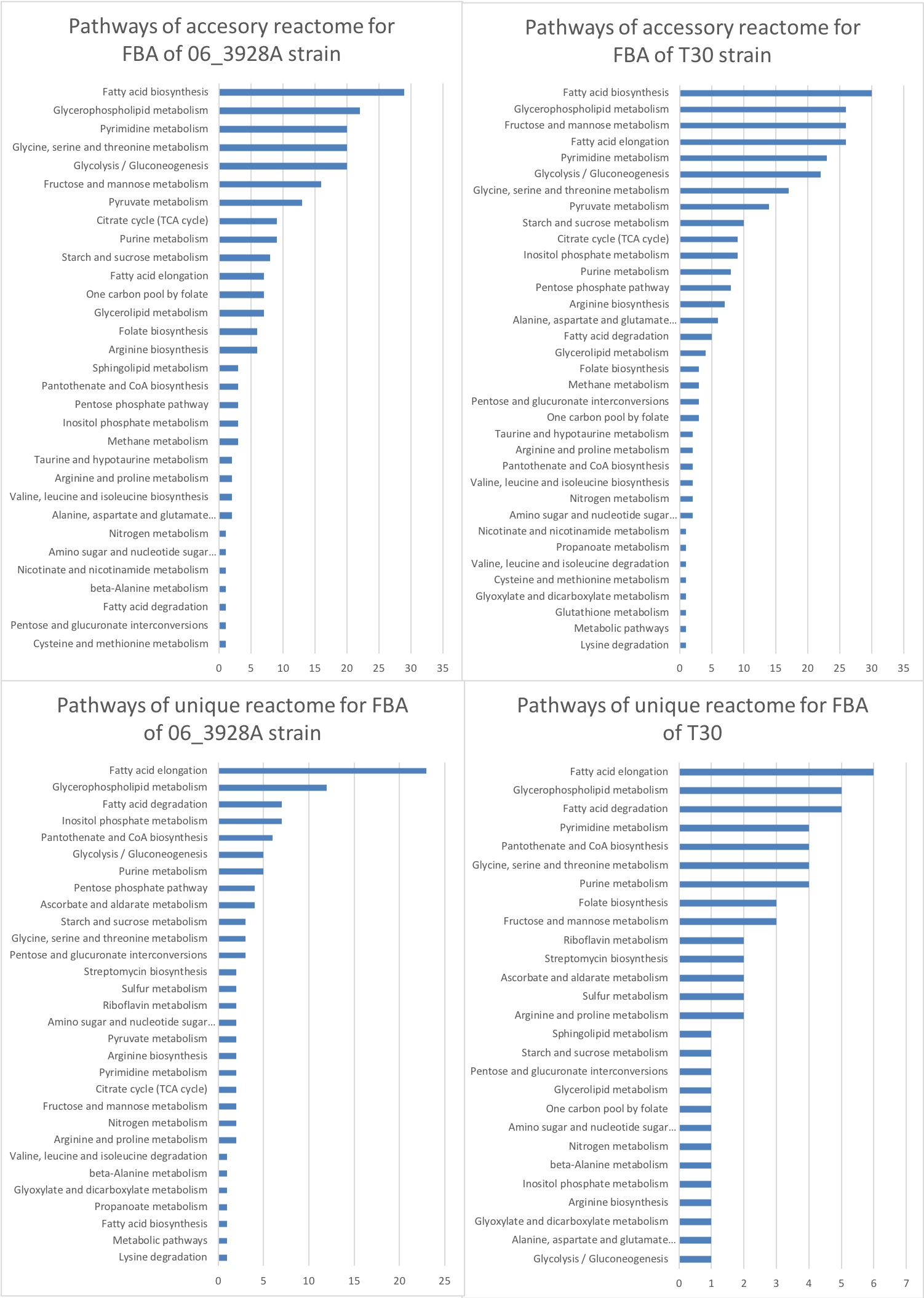

Supplement: Figure S2 — Reactions of the metabolic reconstruction of Phytophthora infestans grouped by metabolic pathways according to the Kyoto Encyclopedia of Genes and Genomes (KEGG) for accessory and unique reactomes calculated over each strain for every time point. [file Image_2.JPEG]

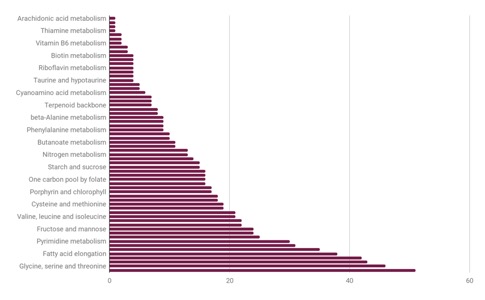

Supplement: Figure S3 — Comparisons between the iSR1301 metabolic model of P. infestans (Rodenburg et al., 2017) and the one presented here at the level of pathways of the reactions shared between the two models. Horizontal axis: number of reactions by pathway. [file Image_3.JPEG]
